# Bioinformatics-based analysis of the lncRNA–miRNA–mRNA and TF regulatory networks reveals functional genes in esophageal squamous cell carcinoma

**DOI:** 10.1042/BSR20201727

**Published:** 2020-08-20

**Authors:** Zhimin Ye, Jun Fang, Zhun Wang, Lei Wang, Bin Li, Tongxin Liu, Yuezhen Wang, Jianfeng Hua, FangZheng Wang, Zhenfu Fu

**Affiliations:** 1 Department of Radiation Oncology, Cancer Hospital of The University of Chinese Academy of Sciences (Zhejiang Cancer Hospital), Banshan East road, Gongshu district, Hangzhou, Zhejiang province, China; 2 Institute of Cancer and Basic Medicine (IBMC), Chinese Academy of Sciences; 3 Key Laboratory of Head & Neck Cancer Translational Research of Zhejiang Province, Banshan East road, Gongshu district, Hangzhou city, Zhejiang province, China; 4 Department of Radiation Oncology, Quzhou Kecheng Hospital, Shuanggang Road, Kecheng district, Quzhou, Zhejiang province, China

**Keywords:** Bioinformatics, ceRNA, core genes, DEGs, Esophageal squamous cell carcinoma, MicroRNAs, Prognosis

## Abstract

Esophageal squamous cell carcinoma (ESCC) is a 5-year survival rate unsatisfied malignancies. The study aimed to identify the novel diagnostic and prognostic targets for ESCC. Expression profiling (GSE89102, GSE97051, and GSE59973) data were downloaded from the GEO database. Then, differentially expressed (DE) lncRNAs, DEmiRNAs, and genes (DEGs) with *P-*values < 0.05, and |log_2_FC| ≥ 2, were identified using GEO2R. Functional enrichment analysis of miRNA-related mRNAs and lncRNA co-expressed mRNA was performed. LncRNA–miRNA–mRNA, protein–protein interaction of miRNA-related mRNAs and DEGs, co-expression, and transcription factors-hub genes network were constructed. The transcriptional data, the diagnostic and prognostic value of hub genes were estimated with ONCOMINE, receiver operating characteristic (ROC) analyses, and Kaplan–Meier plotter, respectively. Also, the expressions of hub genes were assessed through qPCR and Western blot assays. The CDK1, VEGFA, PRDM10, RUNX1, CDK6, HSP90AA1, MYC, EGR1, and SOX2 used as hub genes. And among them, PRDM10, RUNX1, and CDK6 predicted worse overall survival (OS) in ESCC patients. Our results showed that the hub genes were significantly up-regulated in ESCA primary tumor tissues and cell lines, and exhibited excellent diagnostic efficiency. These results suggest that the hub genes may server as potential targets for the diagnosis and treatment of ESCC.

## Introduction

Esophageal cancer (EC) is the eight commonest type of worldwide cancer and the sixth leading gastrointestinal malignancy with a poor survival rate [1], which is caused by many factors, such as tobacco smoking, heavy drinking, lack of fruits and vegetables, and there are two main histological types of EC: esophageal adenocarcinoma (EAC) and esophageal squamous cell carcinoma (ESCC) [2]. ESCC is characterized by weight loss, difficulty in swallowing and a dry cough [3], and accounts for >90% of the cases of EC [4]. Recent years, in particular, have seen major progress in the diagnostic and surgical techniques, radiotherapy, and chemotherapy; however, the improvement of ESCC patient’s survival rates remains unsatisfactory. Hence, identify reliable molecular biomarkers and targets for ESCC early diagnosis and prognosis indication are still urgently needed. Meanwhile, an urgent need for fully comprehensive research into the crucial molecular targets mechanism of ESCC tumorigenesis and metastasis would provide new perspectives into the novel treatment strategy.

Nowadays, long non-coding RNAs (lncRNAs), a class of RNA transcripts with a length of >200 nucleotides that have little or no protein-coding capacity [5,6], have drawn increased attention and accumulating studies demonstrated that lncRNAs take part in multiple biological processes, for example, cell differentiation and proliferation, post-transcriptional and transcriptional modifications, epigenetic modulation, chemotherapy resistance, glucose metabolism, immune response [7,8]. Furthermore, a great many lncRNAs are deregulated in ESCC, making them possibly as potential therapeutic targets or as diagnostic and prognostic biomarkers. MicroRNAs, a kind of small, non-coding RNA, characterized by 20–24 nucleotides in length and play a significant role in gene transcriptional level act as regulators. Abnormal expression of miRNA has been verified to be tightly associated with lncRNAs and transcription factors (TFs), and accumulating researches have indicated that lncRNAs can act as ceRNAs by binding and sequestering the 3′-untranslated region (UTR) of messenger RNAs (mRNAs) to regulate the mRNA and protein expression of target genes [9,10]. lncRNAs and miRNAs are becoming new kinds of diagnostic molecular markers of ESCC, and Chang et al. reported that lncRNA TUSC7 suppressed chemotherapy resistance of ESCC by down-regulating miR-224 to adjust DESC1/EGFR/AKT pathway [11]. Zhong et al. revealed that lnc-ATB promotes malignancy of esophageal squamous cell carcinoma by regulating the miR-200b/Kindlin-2 axis [12]. Tan et al. found lncRNA H19 is overexpressed and promotes ESCC cell proliferation and metastasis [13]. TFs are also as significant factors involved in controlling transcription and post-transcriptional regulation of genes through binding to particular DNA sequences, and involves in proliferation, apoptosis, and migration of tumor cells. Therefore, further study is required to elucidate the interrelationships of lncRNAs, miRNAs, mRNAs, and TFs in ESCC.

With the rapid progress of high-throughput sequencing as well as computational techniques, gene expression profiles analysis contribute to identifying immediately large-scale genes that are differentially expressed at the very same moment, thus more and more lncRNAs, miRNAs, and mRNAs have been discovered [10]. In the present study, we mined the lncRNA, miRNA, and mRNA gene expression data from the recent release of GEO (Gene Expression Omnibus) and TCGA (The Cancer Genome Atlas) database, downloaded the non-coding RNA expression microarray of GSE89102 to identify the differentially expressed lncRNAs (DElncRNAs), and the miRNA expression profile of GSE97051 and GSE59973 to confirm the differentially expressed miRNAs (DEmiRNAs) among ESCC and normal samples. The miRNA-related mRNAs and connected lncRNAs were predicted and then functional enrichment analysis of miRNA-related mRNAs and differentially expressed genes (DEGs) in GSE89102 were also conducted. Besides, lncRNA–miRNA–mRNA network, protein–protein interaction (PPI) network of miRNA-related mRNAs and DEGs, co-expression network of lncRNA and DEGs, regulatory network of transcription factors (TFs) and hub genes were constructed. Importantly, the expression of hub genes was verified by TCGA data. Furthermore, we evaluated the prognostic value of hub genes in patients with EC via ‘The Kaplan-Meier plotter’ database, and also the diagnostic values of hub genes were assessed by receiver operating characteristic (ROC) analyses. As a group, the current research aimed to further explore potential therapeutic targets in the development and progression of ESCC.

## Materials and methods

### Microarray data collection

GEO (http://www.ncbi.nlm.nih.gov/geo/) is a public functional genomics data repository to promote the understanding of gene expression profiles [14]. The long non-coding RNA expression profiles (GSE89102) and two miRNA expression data (GSE97051 and GSE59973) were obtained from GEO (http://www.ncbi.nlm.nih.gov/geo/). The array data of GSE89102 consisted of five paired ESCC tissues and normal tissues based on the platform of GPL16956 (Agilent-045997 Arraystar human lncRNA microarray V3) (Agilent Technologies, Inc., Santa Clara, CA, U.S.A.), deposited by Bai et al. in Third Military Medical University [15]. GSE97051 contained seven paired cancer tissue and adjacent normal tissue (platform: GPL21572 ([miRNA-4] Affymetrix Multispecies miRNA-4 Array) (Affymetrix, Inc., Santa Clara, CA, U.S.A.), and GSE59973 based on the platform of GPL16770 (Agilent-031181 Unrestricted_Human_miRNA_V16.0_Microarray), included three paired of human ESCC tissues and normal controls were submitted by Shi R from the First Affiliated Hospital of Nanjing Medical University.

### Data processing

GEO2R (http://www.ncbi.nlm.nih.gov/geo/geo2r/), an interactive web tool that reprocessed the raw data in a GEO series, also allowing users to evaluate the distribution of the values for the samples have been selected and viewed graphically as a box plot, was employed to identify DElncRNAs, DEmiRNAs and DEGs by comparing ESCC and normal tissue samples. Genes were identified based on the cut off criterion *P*-values *<* 0.05, and |log_2_FC| ≥ 2. The overlapping DEmiRNAs were identified between GSE97051 and GSE59973 through an online tool for Venn diagram analysis (http://bioinfogp.cnb.csic.es/tools/venny/index.html).

### Prediction of target genes and lncRNAs for overlapping DEmiRNAs

The database of miRPathDB [16], targetscan [17], and miRWalk [18] with a score ≥ 0.95 used as a cut-off criterion for the prediction analysis, and was widely used to predict the miRNA-related mRNAs interactions. The interaction between overlapping DEmiRNAs and lncRNAs was predicted by using DIANA-LncBase v2.0 (www.microrna.gr/LncBase), which provides a compositive integration of miRNA–lncRNA interactions [19], with the score≥0.7 as threshold criterion to perform the prediction in the prediction module. No other than miRNA-related mRNAs included in all of miRPathDB, targetscan, and miRWalk databases were picked out for the further functional enrichment analysis and constructed the PPI network. After the predicted lncRNAs were intersected with DElncRNAs in GSE89102, Cytoscape software (version 3.6.1) was utilized to visualize the regulatory network of lncRNA–miRNA–mRNA.

### Integration of protein–protein interaction network and Gene function analysis of selected mRNAs

Search Tool for the Retrieval of Interacting Genes (STRING), a widely used interactive tool (https://string-db.org/) that included known interactions and predicted interactions, was used to explore the interaction of selected mRNAs. Subsequently, PPI pairs that have a combined score≥0.4 (the value was used as a threshold criterion for statistical difference) were identified to construct the network of PPI using Cytoscape. Besides, the selected gene with a degree value ≥5 was identified as hub genes in the PPI network based on an earlier study [10]. Finally, gene ontology (GO) enrichment analyses in terms for biological process (BP), molecular function (MF), and cellular component (CC) for the selected genes were even further conducted with BiNGO tool in Cytoscape [20], and the Metascape (http://metascape.org/) was employed to perform KEGG pathway enrichment analysis [21]. The online tool omicshare (http://www.omicshare.com/) was used to visualize the result of KEGG pathway enrichment analysis by using a bubble diagram.

### Co-expression analysis of selected lncRNAs with DEGs

The GSE89102 also explored the microarray of mRNAs in ESCC. The DEGs with the same criteria for lncRNAs were selected and then a Pearson’s correlation coefficients (PCC) between the selected lncRNAs and DEGs was calculated using the matrix expression in Excel 2010 (Microsoft Corp., Redmond, WA, U.S.A.). 0.95 < PCC < 1 with *P*<0.05 were used as a threshold value to concern significant correlations, and then the genes were chosen to construct the co-expression network with Cytoscape.

We also mined the genes associated with ESCC that proven by literature with PALM-IST. The lncRNA co-expressed DEGs in ESCC-associated gene set were screened out for GO terms as well as KEGG pathways enrichment analysis performed by Metascape, and construction of PPI network also using STRING and visualized by Cytoscape. The terms and pathways with the thresholds were set at a *P*<0.05 and the numbers of enriched genes ≥ 2 [22] were considered as significant to product bubble diagrams. The former five pivotal nodes in the PPI network with degree≥16 were identified to further analysis.

### Prediction of transcription factor for hub DEGs

To explore the interrelation of TFs and hub genes in-depth, TFs of hub genes with adj. *P*<0.05 were investigated by NetworkAnalyst (http://www.networkanalyst.ca), an easy-to-use and high-performance web-based program for biological network analysis, recorded using ChIP Enrichment Analysis (ChEA) [23]. Then, the intergenic interrelation was visualized in Cytoscape and analyzed with a topological property of degree distribution of the network using the tool NetworkAnalyzer.

### TCGA dataset-mining analysis

The TCGA dataset is a platform contains a large cohort of over 30 human tumors for scientists to download and integrated multi-dimensional analyses (https://cancergenome.nih.gov/) [24]. To verify the transcriptional expression of hub genes for improving the reliability of the current study, two online web-based tools, UALCAN (http://ualcan.path.uab.edu/analysis.html), a user-friendly, interactive web resource for analyzing cancer transcriptome data, [25] and Oncomine (www.oncomine.org), a cancer microarray database for contributing to discovery from genome-wide expression analyses [26], were employed to promote data-mining the transcriptional expression level of hub genes. We conducted UALCAN database analysis to assess hub genes expression level based on sample types in TCGA esophageal carcinoma datasets, and transcriptional expression of hub genes for cancer samples compared with those in normal samples on Oncomine database by using Student’s *t*-test with *P*-value < 1E-4 and fold change were defined as 2 for statistically significant, and respectively data type and sample type were selected as mRNA and clinical specimen.

### Survival and ROC analysis of hub genes

Kaplan–Meier plotter (KM plotter, http://kmplot.com/analysis/) is capable to appraise the effect of 54,675 genes on the survival of 10,461 cancer samples [27], and consists of gene expression profiles and survival information for 161 EC patients, was utilized to evaluate the prognostic value of these meaningful hub genes in EC. The OS of EC patients was assessed using a KM plot with patients divided into high and low expression groups according to the median expression of a specific query gene. The hazard ratio (HR) with 95% confidence intervals and log rank *P* values were calculated to assess the relationship of gene expression with survival, and also the number-at-risk is displayed below the curves. Besides, we also performed a ROC analysis with mRNA expression value in GSE89102 to investigate the diagnostic value of hub genes in ESCC using MedCalc software [28].

### Cell culture

Normal esophageal cell lines (HET-1A) and the ESCC cell lines (KYSE-410, KYSE-150, ECA-109, T10, and TE-1) were obtained from the Shanghai Institute of Chinese Academy of Sciences Cell Collection, and cultured in RPMI-1640 medium (Gibco, Grand Island, NY, USA) containing 10% fetal bovine serum (FBS, Gibco), 100 μg/ml streptomycin and 100 U/ml penicillin at 37°C in a 5% CO_2_ incubator.

### qPCR and Western blot analysis

Total RNA was extracted from the cell lines using TRIzol reagent (Invitrogen), and then total RNA was reverse transcribed to complementary DNA (cDNA) by using the PrimeScript RT Reagent Kit (TaKaRa). qPCR was performed using SYBR Premix Ex Taq™ (TaKaRa) on a CFX96 Touch Deep Well detection system (BIORAD, U.S.A.) following the manufacturer’s instructions. GAPDH was used as an internal control. The relative levels of hub genes were estimated using the 2 ^−△△Ct^ method. The primer sequences of hub genes were listed in Table 1.

**Table 1:** The specific primer sequences of hub genes

Gene name	Primer sequence (5′ to 3′)
CDK1	Forward: 5′-GGAAGGGGTTCCTAGTACTGC-3′
	Reverse: 5′-CCATGTACTGACCAGGAGGGA-3′
VEGFA	Forward: 5′-CTGGAGCGTGTACGTTGGT-3′
	Reverse: 5′-TTTAACTCAAGCTGCCTCGC-3′
PRDM10	Forward: 5′-CATCCAGGTCAGCGAGCCTA-3′
	Reverse: 5′-CGAAGTAACCGCCTTCACCT-3′
RUNX1	Forward: 5′-GGGAGCTTGTCCTTTTCCGA-3′
	Reverse: 5′-GAGAGGCAATGGATCCCAGG-3′
CDK6	Forward: 5′-ACAGAGCACCCGAAGTCTTG-3′
	Reverse: 5′-GTATGGGTGAGACAGGGCAC-3′
HSP90AA1	Forward: 5′-CCGCCCAGAGTGCTGAATAC-3′
	Reverse: 5′-ACACCGAACTGGCCAATCAT-3′
MYC	Forward: 5′-CGTCCTCGGATTCTCTGCTC-3′
	Reverse: 5′-GCTGGTGCATTTTCGGTTGT-3′
EGR1	Forward: 5′-GCTGGTGGAGACCAGTTACC-3′
	Reverse: 5′-TCATCGCTCCTGGCAAACTT-3′
SOX2	Forward: 5′-AACCAGCGCATGGACAGTTA-3′
	Reverse: 5′-GACTTGACCACCGAACCCAT-3′
GAPDH	Forward: 5′-GGGAAACTGTGGCGTGAT-3′
	Reverse: 5′-GGGTGTCGCTGTTGAAGT-3′

For Western blot analysis, the cell lines were lysed with RIPA lysis buffer (KenGEN, China), and quantified with a bicinchoninic acid (BCA) kit (Beyotime, China). Equal amounts of cell lysates were separated by 8%, 10% or 12% sodium dodecyl sulfate polyacrylamide gel electrophoresis (SDS-PAGE), and then transferred onto polyvinylidene fluoride (PVDF) membranes (Millipore). After blocking with 5% non-fat milk, the membranes incubated with primary antibodies against CDK1 (1: 2000, ab32094, Abcam), VEGFA (1:2000, ab183100, Abcam), PRDM10 (1:500, Ab3787, Abcam), RUNX1 (1:1000, #4334, Cell Signaling Technology), CDK6 (1:2000, D120398, Sangon), HSP90AA1 (1: 3000, D191063, Sangon), MYC (1:1000, #5605, Cell Signaling Technology), EGR1 (1:2000, ab133695, Abcam), SOX2 (1:1000, #2748, Cell Signaling Technology), GAPDH (1:5000, R1210-1, HuaBio), and β-actin (1:5000, D110001, Sangon) overnight at 4°C. Subsequently, membranes were incubated with appropriate HRP-labeled goat anti-mouse or anti-rabbit secondary antibodies for 2 h at room temperature. After that, the protein bands were visualized with ECL solution (Beyotime, China), and analyzed using ImageJ software (National Institutes of Health, Bethesda, MD).

### Statistical analysis

Correlation between DElncRNAs and DEGs were evaluated using Pearson’s correlation. The Kaplan–Meier survival curves were used to show the differences of hub genes in patient’ OS between the high expression group and low expression group, and the statistical significance was obtained using the two-sided log-rank test. The expression of hub genes in GSE89102 was used to conduct ROC analyses. The difference between the two groups was estimated using a two-tailed Student’s *t*-test, *P*<0.05 was considered statistically significant.

## Results

### Identification of DEmiRNAs, lncRNAs, and mRNAs

The DEmiRNAs were screened using the GEO2R tool. As a result, 26 DEmiRNAs of GSE97051 were identified between ESCC and adjacent normal esophagus samples, including 9 up-regulated and 16 down-regulated DEmiRNAs (Table _1_SuppInfo.xls). About 19 up-regulated and 9 down-regulated DEmiRNAs of GSE59973 also screened out for further analysis (Table _2_SuppInfo.xls).

**Figure 1:**
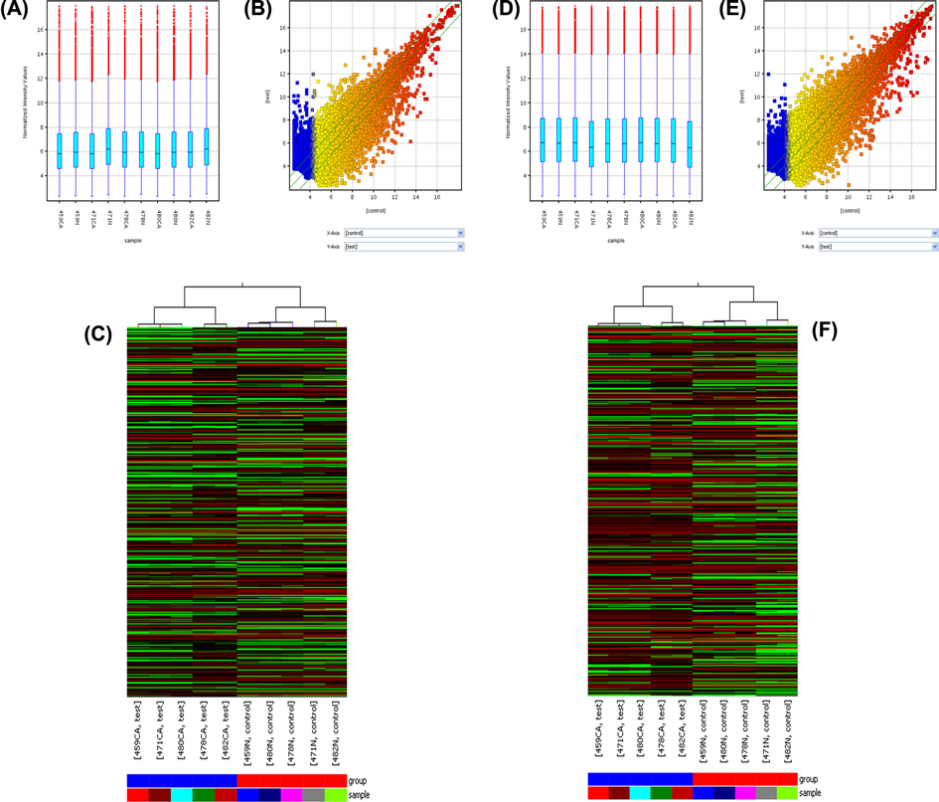
Differentially expressed genes in esophageal squamous cell carcinoma (ESCC) and normal tissue (**A**) Box plot of distributions of expression values of lncRNAs. (**B**) Scatterplot of chips for assessing the variation of lncRNAs. (**C**) Heatmap of lncRNAs. (**D**) Box plot of distributions of expression values of mRNAs. (**E**) Scatterplot of chips for assessing the variation of mRNAs. (**F**) Heatmap of mRNAs.

Meanwhile, the box plot view, scatterplot and hierarchical clustering based on ‘Differentially Expressed LncRNAs’ (Figure 1A–C) of lncRNAs in GSE89102 and the box plot view, scatterplot and hierarchical clustering based on ‘Differentially Expressed mRNAs’ (Figure 1D–F) of mRNAs in GSE89102 were also performed. Both of the box plot shown the distributions of expression values for the samples after normalization, scatterplot assessing the variation (or reproducibility) and hierarchical clustering reveal a differentiable microarray expression profiling among samples. A total of 1583 DElncRNAs comprising 805 up-regulated and 778 down-regulated lncRNAs were obtained (Table_3_SuppInfo.xls). Also, 1916 DEGs, 1330 up-and 586 down-regulated genes (Table _4_SuppInfo.xls) were identified in ESCC compared with normal controls.

### Prediction of target genes and lncRNAs for DEmiRNAs

**Figure 2:**
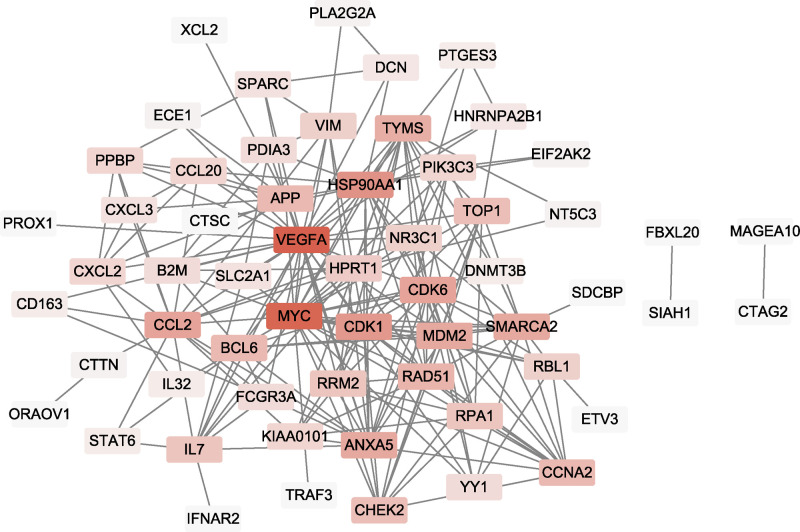
Venn diagram for overlapping DEmiRNAs and overlapping predicted genes (**A**) One DEmiRNAs was up-regulated in both microarrays. (**B**) Two DEmiRNAs were down-regulated in both microarrays. (**C**) 33 predicted genes for hsa-miR-409-3p. (**D**) 1 predicted genes for hsa-miR-133b. (**E**) 44 predicted genes for hsa-miR-139-5p.

Through analysis of microarray data GSE59973 and GSE97051, the up/down-regulated miRNAs were identified. There are three overlapped miRNAs including hsa-miR-409-3p (1 up-regulated, Figure 2A), hsa-miR-133b and hsa-miR-139-5p (2 down-regulated, Figure 2B) in both microarrays. Based on the miRPathDB, miRWalk 2.0, and targetscan database, the relationships between these three DEmiRNAs and predicted genes were identified. The intersection number of these predicted genes for hsa-miR-409-3p was 33 (Figure 2C), for hsa-miR-133b was 1 (Figure 2D) and for the hsa-miR-139-5p was 44 (Figure 2E). A total of 78 predicted genes were listed in Table 2.

**Table 2:** The overlapping genes of selected miRNAs in miRPathDB, miRWalk and Targetscan database

miRNAs	Overlapping genes	Up/Down
hsa-miR-409-3p	MRPL35, PAQR5, AGO2, ZZZ3, SLC25A16, GAB1, TRIM5, C1QTNF1, KLF12, YTHDF3, GPR85, MSRB3, L3MBTL3, ZC3H12C, ADAMTS5, TRIM71, IPO7, NR3C2, DENND2C, MYLK, PHC3, GPR61, SCN1A, CD96, NR4A2, LGSN, TMEM242, LARP1, PAPD5, ZNF423, TNNI1, FAM229B, PRMT6	Up
hsa-miR-133b	RAB5C	Down
hsa-miR-139-5p	AEBP2, ANK2, ARRDC3, ATP11A, ATP2B2, ATXN1, CAMK2D, CCND2, CSRNP2, CTTNBP2NL, DPYSL5, GLCCI1, HEG1, HMGCR, IGF1R, KCND3, KIAA1549, KPNA4, MAP2, MBNL1, NME7, NRK, PDE3A, PDE4A, PGM2L1, PHF6, PIEZO1, PITPNA PMP22, PPARGC1A, PRDM10, PTPN4, RUNX1, SOCS2, STX7, STXBP5L, SYT14, TAOK1, TCF12, TET3, TMPO, TSPAN3, UHMK1, XPO4	Down

Next, the interactions between the three differentially expressed miRNAs and predicted lncRNAs were obtained using LncBase v2.0. dataset. As a result, 535 predicted lncRNAs for hsa-miR-409-3p (Table _5_SuppInfo.xls), 571 predicted lncRNAs for hsa-miR-133b (Table_6_SuppInfo.xls) and 406 predicted lncRNAs for hsa-miR-139-5p (Table _7_SuppInfo.xls) were screened out after deletion of redundant content. Then, the intersection of predicted lncRNAs and up/down regulated DElncRNAs between ESCC and adjacent normal esophagus tissue were performed. Finally, as shown in Table 3. 14 overlapped DElncRNAs for hsa-miR-409-3p, 15 overlapped DElncRNAs for hsa-miR-133b and 9 overlapped DElncRNAs for hsa-miR-139-5p were identified.

**Table 3:** The identified LncRNAs of selected miRNAs in GSE89102

miRNA	Gene Symbol	Location	adj.P.Val	*P*-value	logFC	Down/Up
hsa-miR-409-3P	RP11-1437A8.4	chr16:33203773-33206462	7.87E-03	2.18E-03	-6.478	Down
	RP11-586K12.10	chr16:32663925-32666533	0.00973	0.00288	-5.318	Down
	XLOC_013268	chr19	0.000213	0.000018	-2.054	Down
	LINC00299	chr2:8007771-8324630	0.000766	0.000103	-2.449	Down
	RP11-308D13.3	chr4:139618136-139623232	1.72E-02	5.99E-03	-3.135	Down
	XLOC_004270	chr5	0.00114	0.000174	-2.928	Down
	RMST	chr12:97431653-97565015	0.00798	0.00223	-2.177	Down
	UCA1	chr19:15828961-15836320	3.74E-03	8.36E-04	-2.092	Down
	RP11-214L13.1	chr18:55881688-55920199	3.93E-05	1.71E-06	-2.101	Down
	LINC00475	chr9:92141467-92160114	4.25E-04	4.60E-05	-2.394	Down
	TMEM161B-AS1	chr5:88269024-88436674	1.91E-03	3.49E-04	-3.055	Down
	XLOC_013708	chr20	2.55E-03	5.04E-04	-2.049	Down
	CLDN10-AS1	chr13:95479444-95533910	5.34E-04	6.24E-05	-3.687	Down
	AC104777.3	chr2:150566134-150568080	3.80E-03	8.53E-04	-2.072	Down
hsa-miR-133b	RP11-363G2.4	chr13:22851773-22854138	2.97E-07	7.87E-10	2.287	Up
	THAP9-AS1	chr4:82893009-82900960	1.09E-05	2.70E-07	2.988	Up
	RP11-385J1.2	chr3:178526505-178860352	6.10E-03	1.57E-03	3.327	Up
	AC096559.1	chr2:12576038-12642912	2.98E-03	6.22E-04	2.993	Up
	RP11-562F9.2	chr4:92268767-92277075	1.15E-04	7.80E-06	2.746	Up
	SOX2-OT	chr3:180989783-181699883	6.71E-03	1.78E-03	2.424	Up
	RP11-70C1.1	chr3:42770612-42773635	1.30E-02	4.20E-03	2.096	Up
	DNM3OS	chr1:172136531-172144794	7.19E-03	1.94E-03	2.787	Up
	RP11-657O9.1	chr3:135356067-135439829	1.85E-05	5.72E-07	3.346	Up
	RP11-366F6.2	chrX:151904431-151911455	2.08E-06	1.98E-08	6.928	Up
	LINC00467	chr1:211382803-211435333	3.39E-03	7.38E-04	2.245	Up
	CTB-89H12.4	chr5:149494314-149504670	5.13E-03	1.26E-03	2.999	Up
	RP11-392P7.6	chr12:12927726-12980307	6.86E-03	1.83E-03	2.121	Up
	XLOC_014080	chr21	2.40E-04	2.14E-05	3.045	Up
	RP11-160O5.1	chr17:65100812-65111058	1.30E-04	9.18E-06	2.078	Up
hsa-miR-139-5p	MAST4-IT1	chr5:66662331-66662988	0.0000001	2.23E-10	3.046	Up
	LOC100130744	chr5:14712694-14716529	0.0179	0.00631	2.548	Up
	HCG11	chr6:26523450-26526579	0.000797	0.000108	2.500	Up
	SOX2-OT	chr3:180989783-181699883	0.00671	0.00178	2.424	Up
	XLOC_006242	chr7	0.000583	0.0000706	2.372	Up
	AC009948.5	chr2:178413994-178440243	0.0000728	0.0000041	2.346	Up
	RP11-363G2.4	chr13:22851773-22854138	0.0000002	7.87E-10	2.287	Up
	XLOC_000490	chr1	0.00334	0.000724	2.193	Up
	XLOC_001788	chr2	0.000141	0.0000103	2.088	Up

### Construction of lncRNA–miRNA–mRNA ceRNA and PPI network

**Figure 3:**
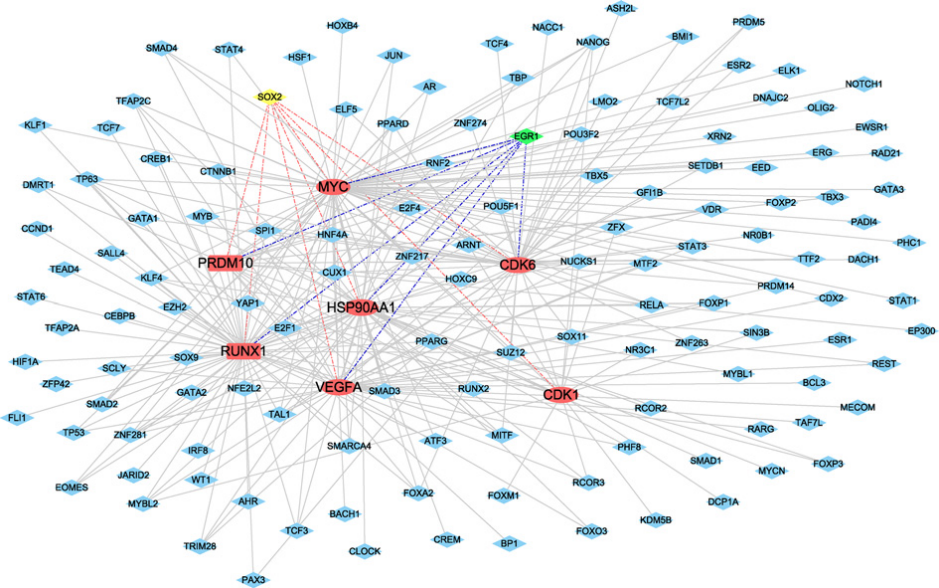
LncRNA–miRNA–mRNA regulatory and PPI networks (**A**) LncRNA–miRNA–mRNA network. Every node represents one gene, and each edge represents the interaction between genes. mRNAs, miRNAs, and lncRNAs are indicated with ellipse, rectangle, and diamond shapes, respectively. Red represents up-regulated and green represents down-regulated. (**B**) PPI network of targets for DEmiRNAs. Nodes represent one gene indicated with a rectangle and continuous mapping node color with brown using degree value.

The aforementioned relationship between miRNA–mRNA and lncRNA–miRNA was used to construct a specific ceRNA (lncRNA–miRNA–mRNA) network using Cytoscape (Figure 3A). We obtained a preliminary understanding of the mechanism of miRNAs, which had a multidimensional regulatory effect through pattern ‘multiple lncRNAs-multiple mRNAs’. According to the information of PPI based on STRING databases, then the PPI network of the 78 predicted genes was constructed (Figure 3B). Additionally, the nodes with a higher value of degree were identified as hub genes. The network consisted of 46 nodes (There is no interaction of 32nodes, which is removed) and 47 edges. The top 2 hub genes included PR/SET domain 10 (PRDM10) and Runt related transcription factor 1 (RUNX1) with the degree of connectivity was 16 and 5, respectively. Combined with the logFC with p-Value of overlapped DElncRNAs, the lncRNA RP11-1437A8.4 and lncRNA MAST4-IT1 were selected for co-expression analysis with down/upregulated DEGs, respectively.

### Functional analysis of the miRNA target gene

**Figure 4:**
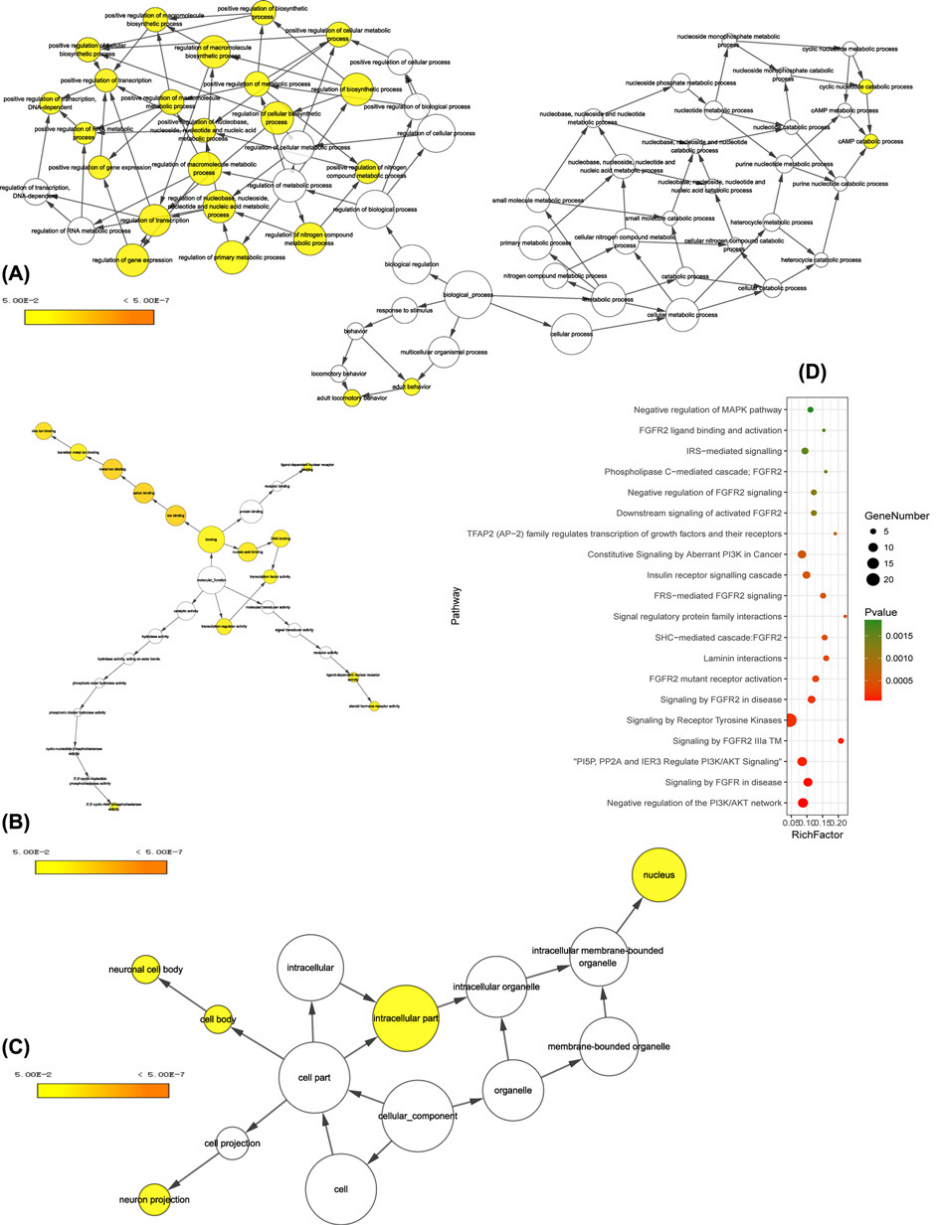
GO terms displayed as an interaction network and KEGG pathways shown as a bubble diagram Yellow nodes: nodes with *P*-value < 0.05 and Benjamini corrected *P*-value <0.05. (**A**) Biological process; (**B**), Molecular function; (**C**), Cellular component; (**D**), KEGG pathways.

To understand the biological functions of predicted genes of DEmiRNAs, GO terms and KEGG pathway analyses of mRNAs in the lncRNA–miRNA–mRNA network were conducted in Cytoscape plug-in BinGO. The results of GO analysis on the BP level (Figure 4A), such as positive regulation of macromolecule biosynthetic process (*P*-value = 3.51E-05, adjusted *P*-value = 1.52E-02), regulation of transcription (*P*-value = 1.17E-04, adjusted *P*-value = 1.52E-02), positive regulation of nitrogen compound metabolic process (*P*-value = 1.50E-04, adjusted *P*-value = 1.52E-02); On the GO terms of MF (Figure 4B), the mRNAs were mainly enriched in metalion binding (*P*-value = 3.47E-06, adjusted *P*-value = 4.61E-04), cation binding (*P*-value = 4.57E-06, adjusted *P*-value = 4.61E-04) and nucleic acid binding (*P*-value = 9.39E-05, adjusted *P*-value = 5.30E-03). On the CC level (Figure 4C), the mRNAs were markedly concentrated in the neuron projection (*P*-value = 1.47E-04, adjusted *P*-value = 3.09E-02), neuronal cell body (*P*-value = 4.52E-04, adjusted *P*-value = 3.16E-02), and nucleus (*P*-value = 6.50E-04, adjusted *P*-value = 3.16E-02). The front 20 most remarkable KEGG pathway terms are displayed, and the mRNAs were primarily concentrating on the Negative regulation of the PI3K/AKT network, Signaling by FGFR in disease and PI5P, PP2A, and IER3 Regulate PI3K/AKT Signaling (Figure 4D).

### Co-expression network of overlapped lncRNAs and DEGs

**Figure 5:**
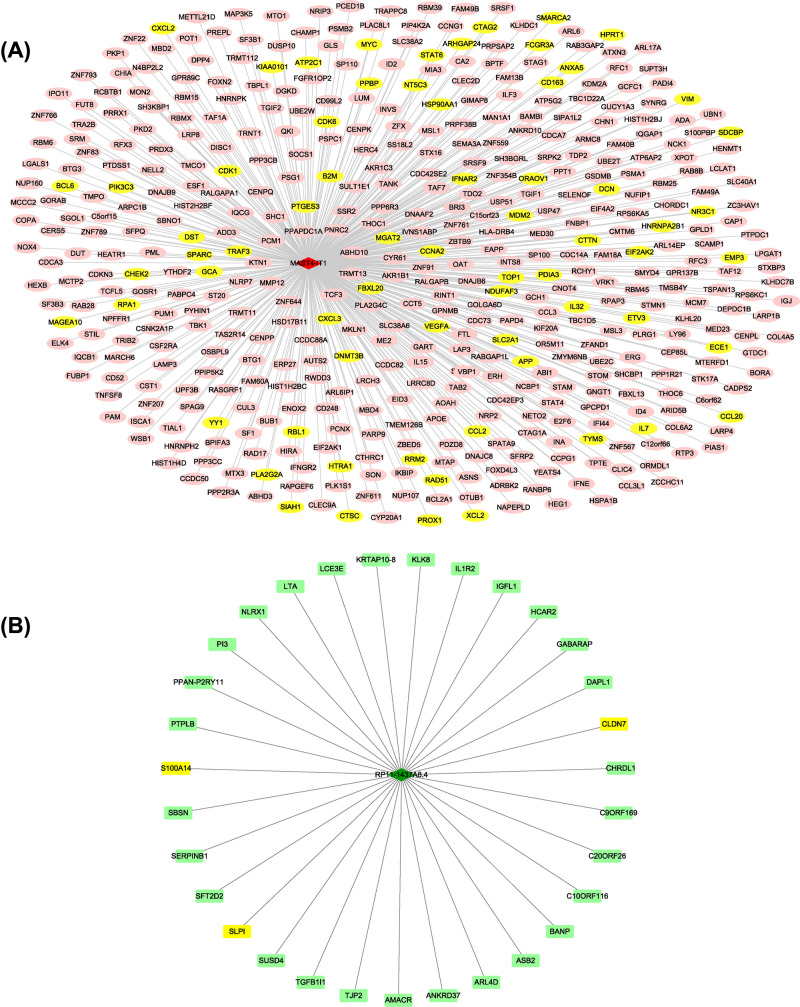
Co-expression network of: (A) lncRNA MAST4-IT1 correlated up-regulated DEGs (*P*<0.05); (B) lncRNA RP11-1437A8.4 correlated down-regulated DEGs (*P*<0.05) The red and bottle green nodes represent lncRNA genes, pink and pale green nodes denote DEGs, respectively. The yellow nodes represent the lncRNA co-expressed mRNAs.

A co-expression network of up/down-regulated DEGs was built up underpinned by their Pearson’s correlation coefficients. The co-expression analysis showed that the lncRNA MAST4-IT1 and 504 up-regulated DEGs (Figure 5A), lncRNA RP11-1437A8.4, and 33 down-regulated DEGs (Figure 5B), whose expressions are significantly correlated. 2481 ESCC-associated genes were mined in PALM-IST. After ESCC-associated genes and co-expressed differentially expressed genes intersecting, 73 the lncRNA co-expressed mRNAs were also screened out in the co-expression network.

### Go and KEGG pathway analysis of the lncRNA co-expressed mRNAs

**Figure 6:**
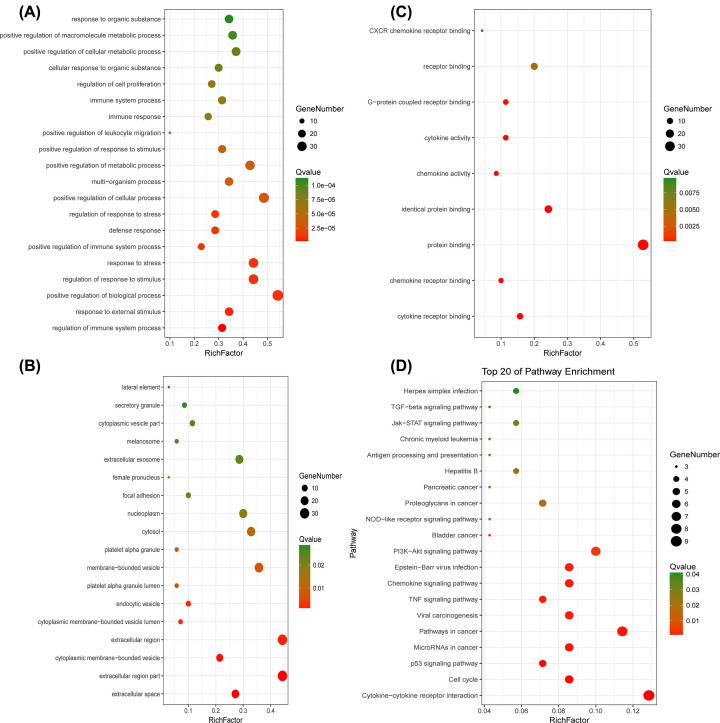
Gene Ontology (GO) and Kyoto Encyclopedia of Genes and Genome (KEGG) enrichment analysis (**A**) The top 20 most significant changes in the GO biological process. (**B**) The top 20 most significant changes in the GO cellular component. (**C**) The top 20 most significant changes in the GO molecular function. (**D**) The top 20 most significant KEGG pathway terms.

To gain further understanding of the bioinformation of identified the lncRNA co-expressed mRNAs, functional enrichment analysis was conducted via Metascape again. The front 20 most important GO terms of each type were shown. The lncRNA co-expressed mRNAs were mainly enriched in regulation of immune system process, response to external stimulus and positive regulation of biological process in biological processes (Figure 6A), related to the extracellular space, extracellular region part and cytoplasmic membrane-bounded vesicle in cellular component (Figure 6B) and also were mainly enriched in cytokine receptor binding, chemokine receptor binding and protein binding on the molecular function level (Figure 6C). The lncRNA co-expressed mRNAs mainly involve in cytokine–cytokine receptor interaction, cell cycle, p53 signaling pathway, microRNAs in cancer and pathways in cancer. The 20 KEGG pathway terms of great significance are also shown in Figure 6D.

### Construction PPI network for the lncRNA co-expressed mRNAs

**Figure 7:**
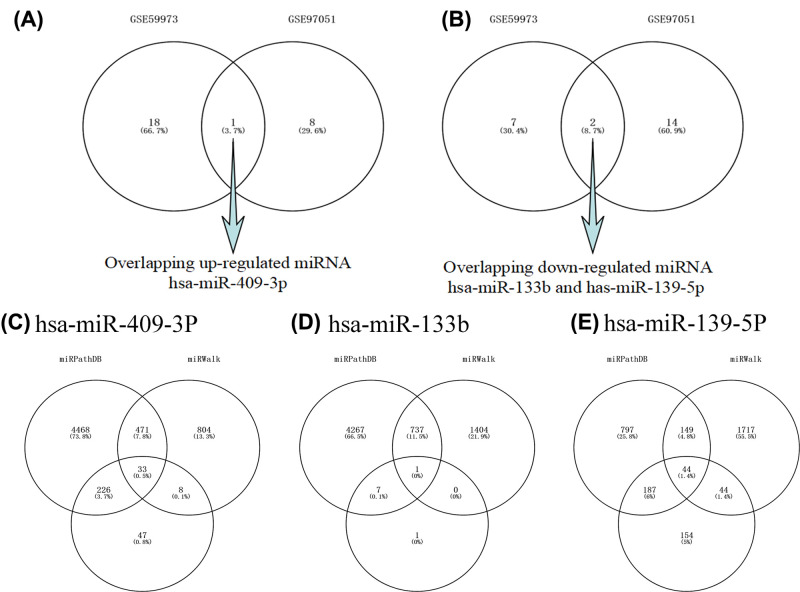
The result of the PPI network with the lncRNA co-expressed mRNAs The node represents the lncRNA co-expressed mRNAs indicated with a rectangle and continuous mapping node color with brown using degree value.

The STRING online database and Cytoscape software were also used to construct the PPI network of the lncRNA co-expressed mRNAs, a total of 70 (except CLDN7, SLPI, and S100A14) of 73 the lncRNA co-expressed mRNAs were mapped into PPI network complex (Figure 7). The GCA, DST, EMP3, MGAT2, HTRA1, NDUFAF3, ATP2C1, ARHGAP24, CLDN7, and SLPI were isolated nodes and then excluded. With degree value>16, 5 nodes were identified as hub genes in the PPI network, including vascular endothelial growth factor A (VEGFA), MYC proto-oncogene, bHLH transcription factor (MYC), heat shock protein 90 alpha family class A member 1 (HSP90AA1), cyclin-dependent kinase 1 (CDK1), and cyclin-dependent kinase 6 (CDK6). These results suggested that the hub genes may significantly play a vital role in the progress of ESCC.

### Transcription factors-hub genes pairs

**Figure 8:**
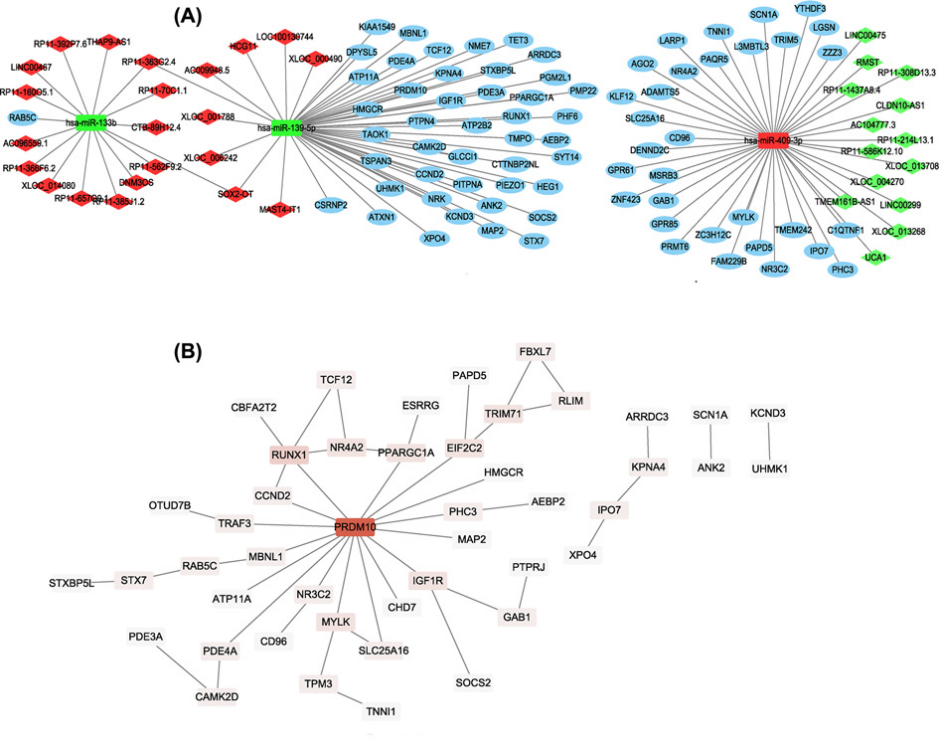
The network of transcription factors and hub genes Nodes represent transcription factors indicated with diamond shapes. Red represents hub genes with an ellipse, light blue represents transcription factors, yellow and green indicated that the TFs regulating 7 or 6 hub genes, respectively.

As shown in Figure 8, the transcription-regulated network with 141 nodes and 296 edges, and multiple hub genes associated with TFs are shown in Table 4. Interestingly, we realized that SRY (sex determining region Y)-box 2 (SOX2) had been predicted to regulate CDK1, VEGFA, PRDM10, RUNX1, CDK6, HSP90AA1 and MYC, 7 hub genes, and MYC, RUNX1, CDK6, HSP90AA1, VEGFA, and PRDM10 could be regulated by early growth response 1 (EGR1).

**Table 4:** The transcription factors of hub genes

TFS	Genes	Total
SOX2	MYC RUNX1 CDK6 HSP90AA1 VEGFA PRDM10 CDK1	7
EGR1	MYC RUNX1 CDK6 HSP90AA1 VEGFA PRDM10	6
TP63	MYC RUNX1 CDK6 VEGFA CDK1	5
NUCKS1	MYC CDK6 HSP90AA1 VEGFA CDK1	5
NANOG	MYC CDK6 HSP90AA1 PRDM10 CDK1	5
TFAP2C	MYC RUNX1 CDK6 HSP90AA1 VEGFA	5
TAL1	MYC RUNX1 CDK6 HSP90AA1 VEGFA	5
ZNF281	MYC RUNX1 CDK6 HSP90AA1 VEGFA	5
HNF4A	MYC RUNX1 CDK6 VEGFA PRDM10	5
E2F4	MYC RUNX1 CDK6 HSP90AA1 PRDM10	5
PPARG	MYC RUNX1 HSP90AA1 VEGFA CDK1	5
E2F1	MYC RUNX1 HSP90AA1 PRDM10 CDK1	5
MYBL2	RUNX1 CDK6 HSP90AA1 CDK1	4
TRIM28	RUNX1 CDK6 HSP90AA1 VEGFA	4
MITF	RUNX1 CDK6 HSP90AA1 PRDM10	4
FOXA2	RUNX1 CDK6 HSP90AA1 PRDM10	4
POU5F1	MYC CDK1 CDK6 HSP90AA1	4
SPI1	MYC RUNX1 CDK6 HSP90AA1	4
CUX1	MYC RUNX1 CDK6 HSP90AA1	4
MYB	MYC RUNX1 CDK6 HSP90AA1	4
KLF4	MYC RUNX1 CDK6 HSP90AA1	4
PPARD	MYC RUNX1 CDK1 HSP90AA1	4
SMAD2	MYC RUNX1 HSP90AA1 PRDM10	4
SMAD3	MYC RUNX1 PRDM10 HSP90AA1	4
MTF2	RUNX1 CDK6 VEGFA	3
RCOR3	HSP90AA1 VEGFA CDK1	3
STAT3	MYC CDK6 VEGFA	3
SMAD4	MYC CDK6 VEGFA	3
PRDM14	MYC CDK6 VEGFA	3
ZNF217	MYC RUNX1 CDK6	3
SUZ12	MYC RUNX1 CDK6	3
CTNNB1	MYC RUNX1 CDK6	3
RELA	MYC CDK6 HSP90AA1	3
ELF5	MYC RUNX1 CDK6	3
RUNX2	RUNX1 VEGFA PRDM10	3

### Dysregulated expression of hub genes in patients with ESCC

**Figure 9:**
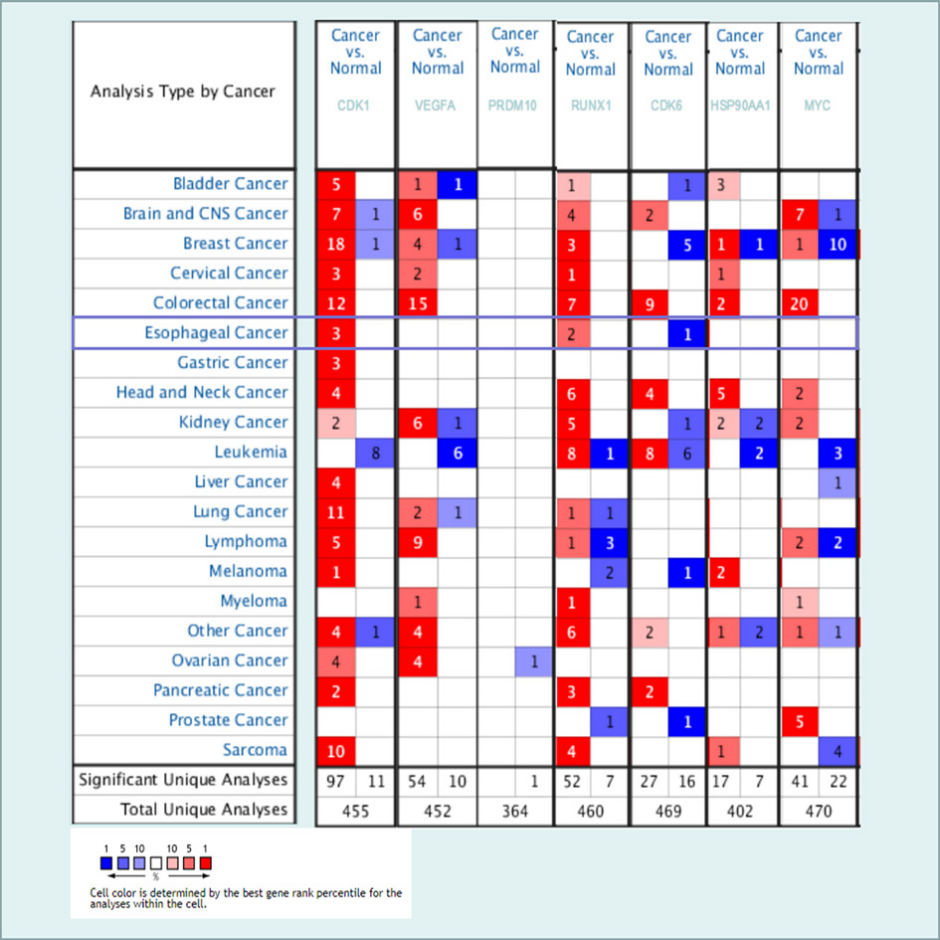
Transcriptional levels of 7 hub genes in different types of cancers This information was attained from Oncomine with the threshold of *P*-value ≤ 1E-4, Fold Change≥2, and Gene Rank ≥Top10% and indicates the numbers of datasets with statistically significant (*P*<0.05) mRNA high-expression (red) or low-expression (blue) of CDK1, VEGFA, PRDM10, RUNX1, CDK6, HSP90AA1, and MYC (different types of cancer vs. corresponding normal tissue). Cell color was decided by the best gene rank percentile for the analyses within the cell, and the gene rank was analyzed by percentile of target genes at the top of all genes measured by each study.

**Figure 10:**
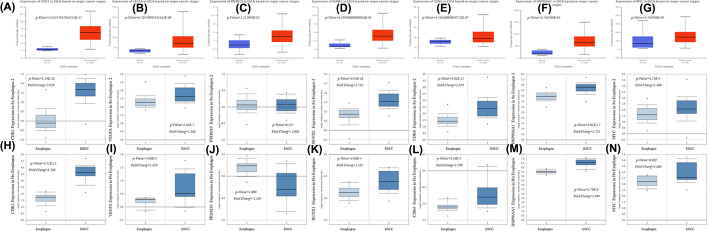
TCGA data portal analysis using UALCAN and Oncomine database (**A–G**) Hub gene CDK1, VEGFA, PRDM10, RUNX1, CDK6, HSP90AA1, and MYC expression in EC. (**H–N**) Hub gene CDK1, VEGFA, PRDM10, RUNX1, CDK6, HSP90AA1, and MYC expression based on Oncomine for ESCC.

Seven hub genes were observed using the UALCAN and Oncomine database. As a preliminary step, we measured the expression of CDK1, VEGFA, PRDM10, RUNX1, CDK6, HSP90AA1, and MYC in 20 different kinds of cancer, and compared with normal individuals as shown in Figure 9. The mRNA expression of CDK1 and RUNX1 was significantly raised in EC samples in multiple datasets. TCGA dataset mining was performed using UALCAN to shown that abnormal expression of the hub genes, including CDK1, VEGFA, PRDM10, RUNX1, CDK6, HSP90AA1, and MYC, were significantly up-regulated between ESCA primary tumor and normal tissues (Figure 10). Similarly, the hub genes CDK1, VEGFA, RUNX1, CDK6, HSP90AA1, and MYC with fold change 2.929, 1.368, 1.733, 1.929, 1.724, and 1.408, respectively in Su Esophagus 2 dataset [29] (Figure 10). Besides, the hub genes CDK1, VEGFA, RUNX1, CDK6, HSP90AA1, and MYC with fold change 3.760, 1.420, 1.182, 1.299, 1.994, and 1.604, respectively in Hu Esophagus [30] dataset (Figure 10). Both sources report markedly increased expression of 6 hub genes were associated with ESCC, in contrast to normal tissue, excluding PRDM10.

### The diagnostic, prognostic value, and expression levels of hub genes in EC

**Figure 11:**
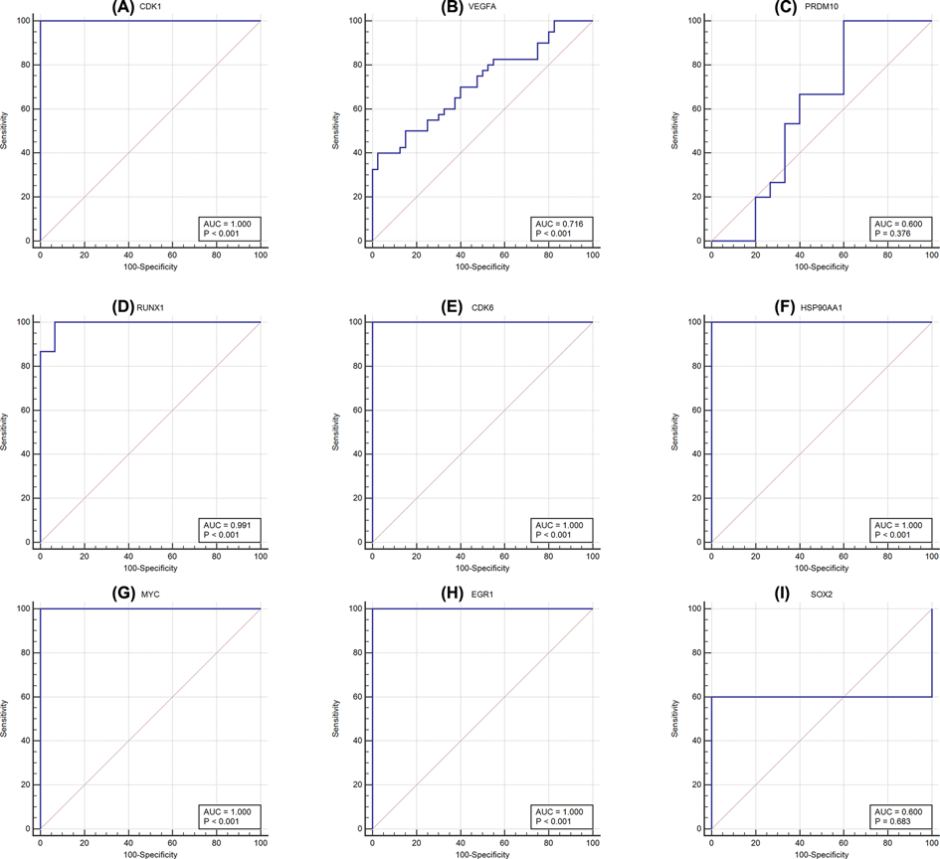
The receiver operating characteristic (ROC) curves of the nine hub genes from PPI and TFs networks (**A**) CDK1; (**B**) VEGFA; (**C**) PRDM10; (**D**) RUNX1; (**E**) CDK6; (**F**) HSP90AA1; (**G**) MYC; (**H**) EGR1; (**I**), SOX2.ROC curves were drawn using MedCalc software. AUC: area under the ROC curve.

The ROC analysis of the nine hub genes expression of CDK1, VEGFA, PRDM10, RUNX1, CDK6, HSP90AA1, MYC, EGR1, and SOX2 from PPI and TFs networks was performed. The results indicated that the areas under curves (AUC) for CDK1, RUNX1, CDK6, HSP90AA1, MYC, and EGR1 reached 1 with *P*<0.001, respectively, and 0.716 with *P*<0.001 for VEGFA (Figure 11). All of them may be provided a potential diagnostic efficiency in the future for clinical relevance, excluding PRDM10 (AUC = 0.600, *P*=0.376) and SOX2 (AUC = 0.600, *P*=0.683) in these experimental conditions.

**Figure 12:**
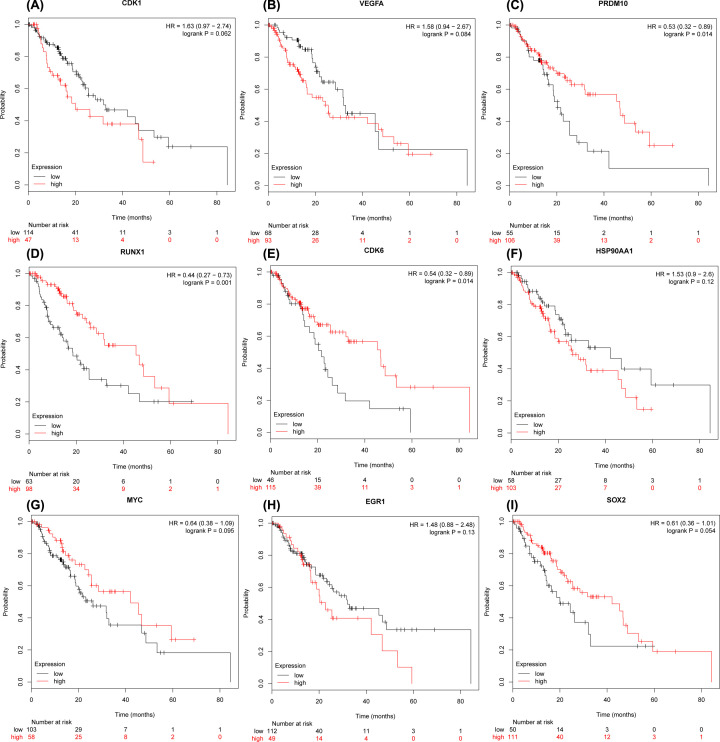
Prognostic value of nine hub genes from PPI and TFs networks (**A**) CDK1; (**B**) VEGFA;(**C**) PRDM10; (**D**) RUNX1; (**E**) CDK6; (**F**) HSP90AA1; (**G**) MYC; (**H**) EGR1; (**I**), SOX2. HR, hazard ratio; CI, confidence interval.

**Figure 13:**
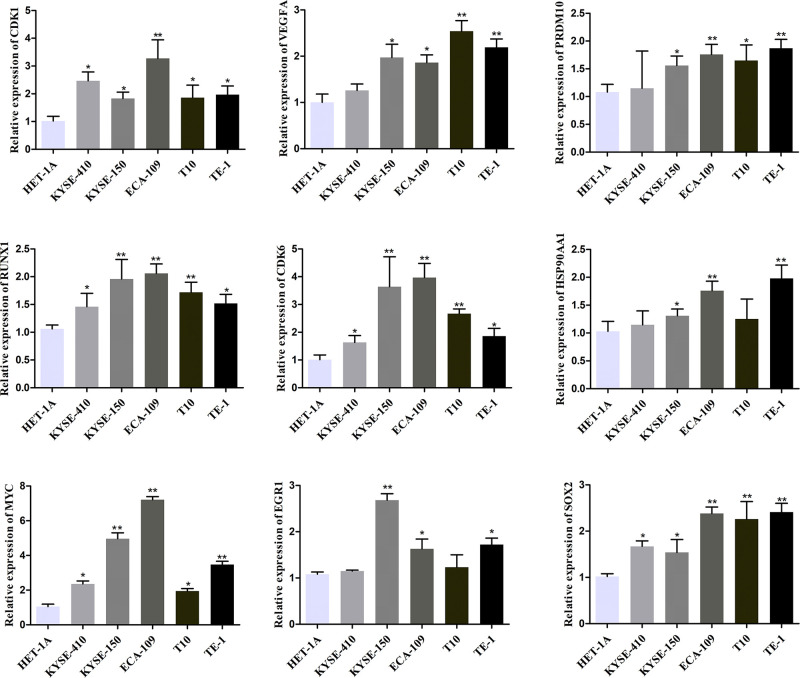
mRNA expression levels of hub genes in ESCC cell lines The mRNA expression levels of CDK1, VEGFA, PRDM10, RUNX1, CDK6, HSP90AA1, MYC, EGR1, and SOX2 were examined by qPCR. Each bar depicts the mean values (mean ± s.d.); *n*=6 per group; *P<0.05; **P<0.01 vs. HET-1A cells.

**Figure 14:**
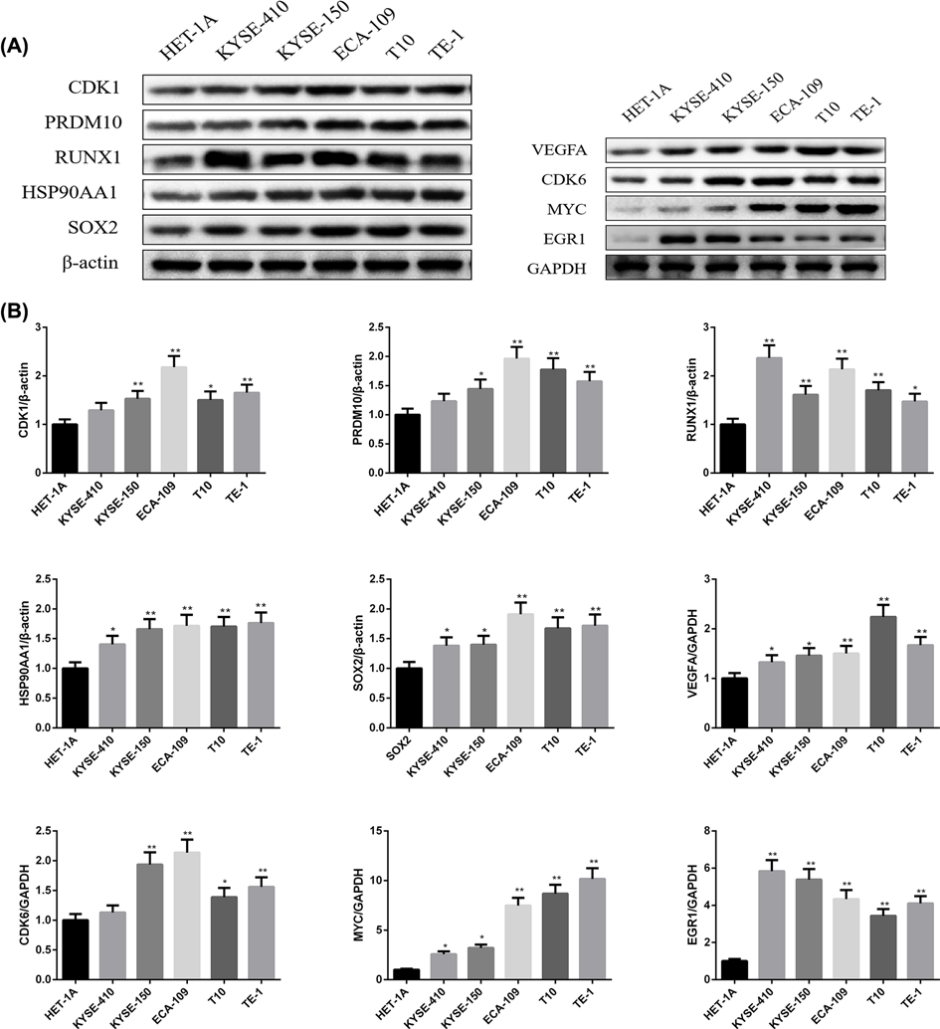
Protein expression levels of hub genes in ESCC cell lines (**A**) Evaluation of CDK1, VEGFA, PRDM10, RUNX1, CDK6, HSP90AA1, MYC, EGR1, and SOX2 protein expression by Western Blot. (**B**) Representative Western blot analysis of CDK1, VEGFA, PRDM10, RUNX1, CDK6, HSP90AA1, MYC, EGR1, and SOX2 in the ESCC cell lines. Each bar depicts the mean values (mean ± S.D.); *n*=3 per group. *P<0.05; **P<0.01 vs. HET-1A cells.

The prognostic value of nine hub genes CDK1, VEGFA, PRDM10, RUNX1, CDK6, HSP90AA1, MYC, EGR1, and SOX2 from PPI, and TFs regulated networks were obtained from KM plotter. It was realized that low transcription level of PRDM10 (HR, 0.53; 95% confidence interval [CI], 0.32–0.89; *P*=0.014), RUNX1 (HR, 0.44; 95% CI, 0.27–0.73; *P*=0.001) and CDK6 (HR, 0.54; 95%CI, 0.32–0.89; *P*=0.014) were associated with worse OS for EC patients; however, the CDK1, VEGFA, HSP90AA1, MYC, EGR1 and SOX2 were not different significantly (*P*>0.05) between high and low expression groups in Figure 12. The expression levels of hub genes in ESCC cell lines were measured using qPCR and Western blot. As shown in Figures 13 and 14 (Supplementary Figure S1). Compared with HET-1A cells, the mRNA and protein expression of CDK1, VEGFA, PRDM10, RUNX1, CDK6, HSP90AA1, MYC, EGR1, and SOX2 in ESCC cell lines was significantly up-regulated to a certain extent. These findings collectively indicate that hub genes may be involved in ESCC.

## Discussion

Abnormal expression and transcription levels of miRNAs and lncRNAs have been demonstrated related to disease progression in esophageal carcinogenesis and have great potential as a noninvasive biomarker for cancer diagnosis and prognosis [31]. Despite the number of coding genes that have been determined playing a fundamental role in the gene expression regulation for ESCC development, and an early diagnosis for ESCC patients who have will obtain longer survival times. However, the pathogenesis of the disease remains poorly investigate, and much of the change in gene expression and biological marker associated with ESCC remains to be fully elucidated. Previous studies have reported multiple candidate miRNAs and lncRNAs for ESCC detection, treatment, and prognosis. MiR-99a down-regulated in ESCC significantly promoted tumor cell proliferation, migration, invasion, and slug-induced EMT through activating the IGF1R signaling pathway [32]. Mei et al. reported that overexpression of miR-125b-5p inhibited cell proliferation, migration, and invasion partially by down-regulating HMGA2 in ESCC [33]. Zhao et al. found that miR-543 was higher expression and down-regulated expression of PLA2G4A in ESCC cells with enhanced cell invasiveness and mobility [34]. Long non-coding RNA DANCR was up-regulated, similar to an oncogene, and knocked down the expression of DANCR significantly suppressed cell migration, proliferation, and invasion in human ESCC tissues [35]. Liang et al. demonstrated that the up-regulated expression of LncRNA CASC9 that connected with ESCC metastasis and prognosis, indicating that CASC9 could use as a targeted therapeutic strategy for the treatment of metastasis [36]. In our study, we reanalysis the miRNA and lncRNA dataset from the GEO dataset for biomarker discovery using comprehensive bioinformatics analysis. As a result, the miRNAs (miR-409-3p, miR-133b, and miR-139-5p) and lncRNAs (MAST4-IT1, RP11-1437A8.4) were identified for deeper functional analysis, which may also have an important effect on the progress of ESCC.

miR-409 serves as a newly discovered manager in multiple types of cancer which directly targeting radixin to depress tumor cell metastasis and invasion in gastric cancer [37], also functions as a tumor suppressor in non-small-cell lung cancer (NSCLC) through PI3K/AKT pathway by directly targeting SPIN1 [38]. Besides, overexpression of miR-409-3p has the power to enhance the chemosensitivity of colon cancer cells by depressing Beclin-1-induced autophagy [39]. miR-133b, located on chromosome 18, has been commonly identified as being down-regulated in ESCC. Huang et al. reported that miR-133b was noticeably descended in ESCC tumor tissues and all kinds of ESCC cell lines, and the overexpression of miR-133b remarkably contribute to inhibit the proliferation and advance the apoptosis of Eca-109 and KYSE150 cells by targeting cullin 4B [40]. Wu et al. [41] and Kano et al. [42] also unanimously reported that expression of miR-133b was down-regulated, and Kano further found that miR-133b inhibits cell proliferation and cell invasion in ESCC cells directly regulate FSCN1 expression. A study found that miR-139-5p has the tumor-suppressive features, invasiveness, and growth suppressing characteristic in human ESCC by targeting the 3′ UTR of oncogenic NR5A2 [43]. Additionally, Yang et al. demonstrated that down-regulation of hsa-miR-139-5p when in comparison with control samples using miRNA microarrays, and increased the risk of EC [44]. Yet, the accurate function of miR-409 in human ESCC still largely undefined, which needed more experiments to further explore. Consistent with the results from previous studies, in the present study, we also found that miR-409-3p was up-regulated, miR-133b and miR-139-5p were down-regulated in both GSE97051and GSE59973 dataset. The functional enrichment analysis of miRNAs-target indicated that DEmiRNAs associated with negative regulation of the PI3K/AKT network pathway. In addition, high mRNA expression of miR-409 and miR-139-5p were associated with worse OS for EC patients, yet have no statistical difference, including hsa-miR-133b (Table _8_SuppInfo.xls). Therefore, the results are in accordance with these previous studies, which suggests that hsa-miR-133b and hsa-miR-139-5p probably indirectly or directly play important roles in ESCC development.

Long non-coding RNAs have been discovered to be special molecular regulators in ESCC. Previous studies have shown that overexpression of lncRNA GAS5 would result in cell cycle arrest at the G2/M stage by regulating the ATM-CHK2 pathway to inhibit cell proliferation, migration, and invasion in ESCC [45]. Similarly, a study has found that positive expression of LncRNA FER1L4 inhibits cell proliferation, invasion, promotes apoptosis and increases the cell cycle distribution in G0/G1 [46]. In the present study, significant lncRNA MAST4-IT1 (ENST00000514241) up-regulation and lncRNA RP11-1437A8.4 (ENST00000565847) down-regulation were also observed in ESCC tissue compared with control tissue in GSE89102. The differentially expressed lncRNAs and 70 potential correlative mRNA targets were then integrated into the lncRNA and DEGs co-expression network, and bioinformatic analysis demonstrated that these dysregulated lncRNAs are connected with cell cycle, p53 signaling pathway, MicroRNAs in cancer and pathways in cancer. In brief, our results revealed the DElncRNAs have a probable role in ESCC development and progression. On the other hand, 7 hub genes (CDK1, VEGFA, PRDM10, RUNX1, CDK6, HSP90AA1, and MYC) were up-regulated in ESCC tissues in comparison with normal tissues in the PPI network. Meanwhile, CDK1, RUNX1, CDK6, HSP90AA1, MYC, VEGFA, and TFs, EGR1 (excluding SOX2), may probably be used as promising diagnostic biological targets for ESCC, and the highly increased expression level of PRDM10, RUNX1 and CDK6 is closely connected with the better OS of this cancer, which also potential identified as a promising prognostic factor in ESCC.

As we know that, G2/M phase cell cycle arrest resulting in a reduction of proliferation and improve apoptosis [47], and Cyclin-dependent kinase 1 (CDK1, G2/M phase transition-related gene) and Cyclin-dependent kinase 6 (CDK6, G1-S phase-related gene) is also important to regulate cell cycle progression [48,49]. Numerous studies have demonstrated that increasing the cells in the G2/M cell cycle contributes to the radiosensitivity of many malignant tumors, including ESCC, by down-regulating CDK1 expression [48]. CDK4 and CDK6, being highly homogenous, can be bound and activated by the D-type Cyclins (Cyclin D1, D2, and D3). Aberrant activation of the Cyclin D1-CDK4/6-Rb signaling pathway is common in ESCC, suggesting that CDK6 amplification promoting the progress of aggressive type of esophageal cancer. Interestingly, the OS of a high expression of CDK6 indicated a better prognosis of ESCC patients in the present study. So, the roles of CDK6 need to be further investigated. Vascular endothelial growth factor (VEGF) family, produced by tumor cells, macrophages, platelets, stroma, and other host cells, the most prominent stimulating angiogenic factor, playing in tumor cell differentiation, the promotion of tumor cell migration and invasion [50]. VEGFA is one of the essential growth factors for tumor angiogenesis in endothelial cells, and higher VEGFA expression as a marker indicating that an advanced stage with a poor OS for patients with esophageal cancer [51]. In the present study, the OS of VEGFA in the high expression group, as analyzed in the Kaplan–Meier plotter, revealed the same trend. PRDM10 is a member of the PRDM family of PRDI-BF1 and RIZ homology domain-containing proteins [52] which might play an essential role in gene expression but is poorly studied in ESCC so far. Although several other members of the PRDM family are associated with cancer especially the PRDM16. The high expression of PRDM10 has a superior OS than low expression, and revealed that PRDM10 could potentially be a prognosticator. RUNX1, the predominant RUNX family member, which has central roles in both neoplasia and normal development [53], also potentially functions as tumor oncogenes or suppressors on the basis of cellular context. Besides, it has been reported that lincRNA-uc002yug.2 contributes to a combination of alternative splicing (AS) factors and RUNX1 to promote ESCC progression, and this suggested that RUNX1 has a tumor-suppressing role in esophageal tumors [54]. We also speculated that the lncRNA MAST4-IT1/has-miR-139-5p/RUNX1 axis was correlated with all aspects of ESCC. Heat shock protein 90 alpha family class A member 1 (HSP90AA1), also known as HSP90α, localized in the cytoplasm and played an important role in the proper folding, assembly and localization of many cellular proteins, which required for tumor progression and highly expressed in many cancer cells, inhibition of HSP90AA1 is a promising strategy for cancer therapy [55,56]. Similarly, a study has found that up-regulated HSP90AA1 mRNA expression indicates poor prognosis of HER2 negative breast cancer patients [57]. MYC is a highly pleiotropic DNA-binding transcription factor [58], and high expression of MYC is extensively implicated in promoted tumor growth, tumorigenesis, and drug resistance in diverse human cancers [59]. Moreover, some studies found that suppressing MYC is associated with radiosensitizes and attenuates malignant growth in ESCC [59,60]. EGR1, a tumor suppressor, which down-regulated in ESCC tissues is involved in tumorigenesis and results in enhancive tumor transformation [61]. A study found that the silencing of EGR1 reduced cisplatin-induced apoptosis in WHCO1 cells [62]. Additionally, Gao et al. also reported that miR-191 could increase cell proliferation and invasion, which might reduce EGR1 expression by binding its 3′UTR involved in ESCC progression [63]. SOX2 was the first one identified, located at chromosome 3q26.33, as a 317 amino acid transcription factor containing an HMG domain, which involved in cell proliferation, apoptosis, differentiation and affects the prognosis of ESCC [64]. Similarly, Wang et al. further demonstrated that high expression of SOX2 in ESCC tissues and the expression was observably related to N stage (*P*=0.034), differentiation (*P*=0.003), and also had remarkably better survival benefit than those with low SOX2 expression (*P*=0.021) [65], which consistent with the current research. Significantly, the expression of hub genes remarkably increased in ESCC cells, as confirmed by qPCR and Western blot assays.

In summary, the present study was intended to identify the potential molecular target and analyze its prognostic values in ESCC by bioinformatics analysis. As a result, we identified that hub gene, CDK1, VEGFA, PRDM10, RUNX1, CDK6, HSP90AA1, MYC, EGR1, and SOX2 from PPI and TFs networks. Additionally, the differential expression of these hub genes was also confirmed at transcriptional and protein levels using qPCR and Western blot in the ESCC cells, indicating that these targets have close links of ESCC development and growth. However, we realized that the present study was performed by bioinformatics analysis and the results, in part, does not cohere with other studies. For this reason, the limitations of our study are the data used were only accessed from the public database. Therefore, further functional investigations of these targets are essential.

On the whole, our findings demonstrate that hub genes, CDK1, VEGFA, PRDM10, RUNX1, CDK6, HSP90AA1, MYC, EGR1, and SOX2 regulates ESCC cells via multiple networks involved in various signaling contexts, which would provide novel therapeutic strategies for ESCC patients.

## Supplementary Material

Supplementary Figure S1

Supplementary Tables S1-S8

## Abbreviations

AS: alternative splicing
BP: biological process
CC: cellular component
CDK1: Cyclin-dependent kinase 1
ChEA: ChIP Enrichment Analysis
DEG: differentially expressed gene
DElncRNA: differentially expressed lncRNA
DEmiRNA: differentially expressed miRNA
EAC: esophageal adenocarcinoma
EC: esophageal cancer
ESCC: esophageal squamous cell carcinoma
GEO: Gene Expression Omnibus
GO: Gene Ontology
HR: hazard ratio
HSP90AA1: Heat shock protein 90 alpha family class A member 1
KM plotter: Kaplan–Meier plotter
lncRNAs: long non-coding RNA
MF: molecular function
mRNA: messenger RNA
OS: overall survival
PCC: Pearson’s correlation coefficients
PPI: protein–protein interaction
ROC: receiver operating characteristic
STRING: Search Tool for the Retrieval of Interacting Genes
TCGA: The Cancer Genome Atlas
TF: transcription factor
UTR: 3′-untranslated region
VEGF: vascular endothelial growth factor
