# Supplementary figures and images for: Bioinformatics-based analysis of the lncRNA–miRNA–mRNA and TF regulatory networks reveals functional genes in esophageal squamous cell carcinoma
Source: Biosci Rep. 2020 Aug 20;40(8):BSR20201727. doi: 10.1042/BSR20201727 (PMC7441485; doi:10.1042/BSR20201727)

**Supplementary Figure S1**  
**Full unedited blots for Figure 14A**

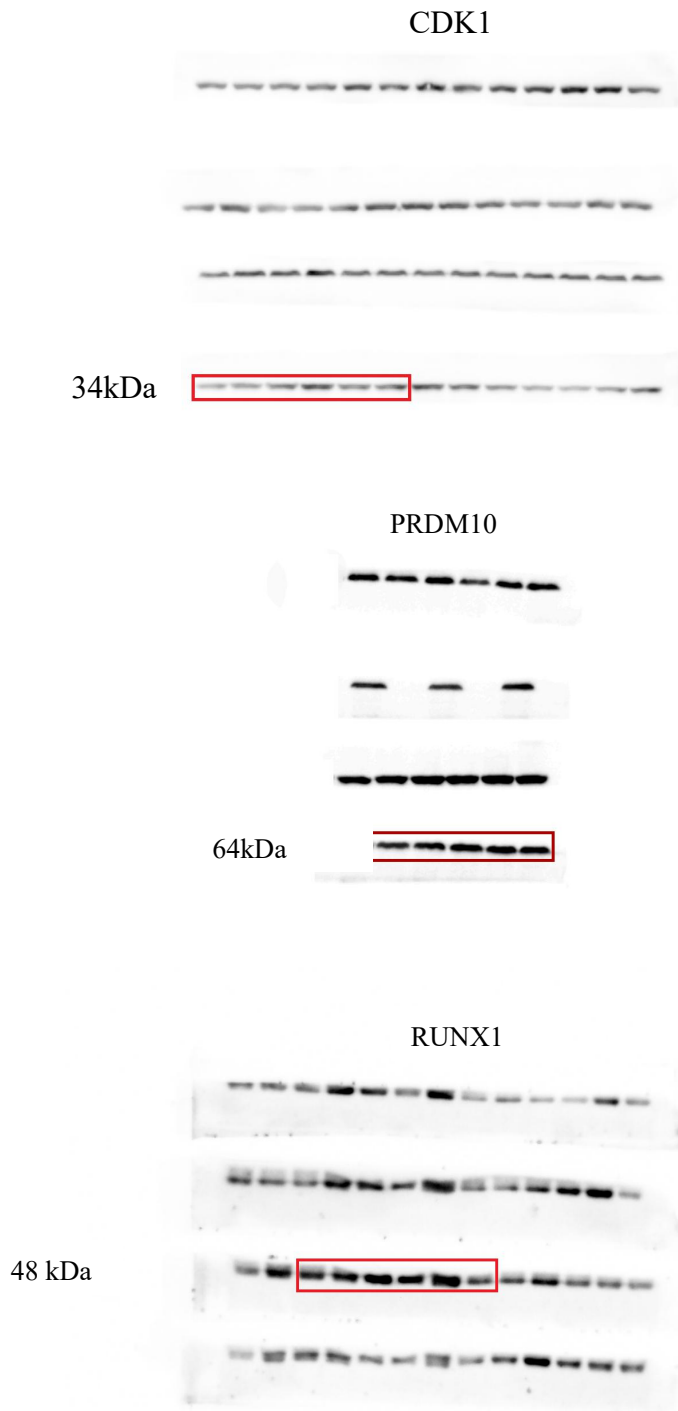

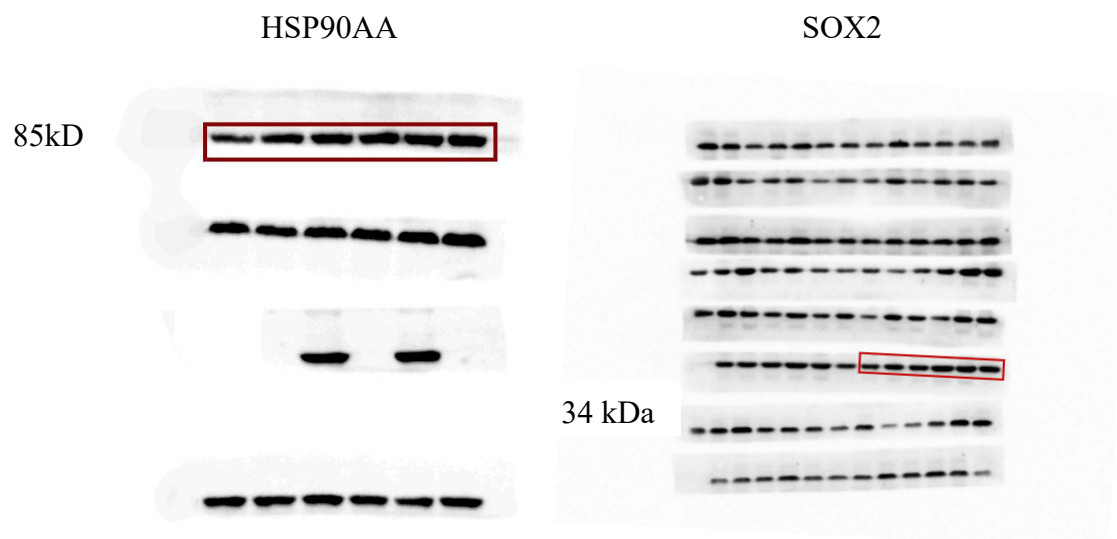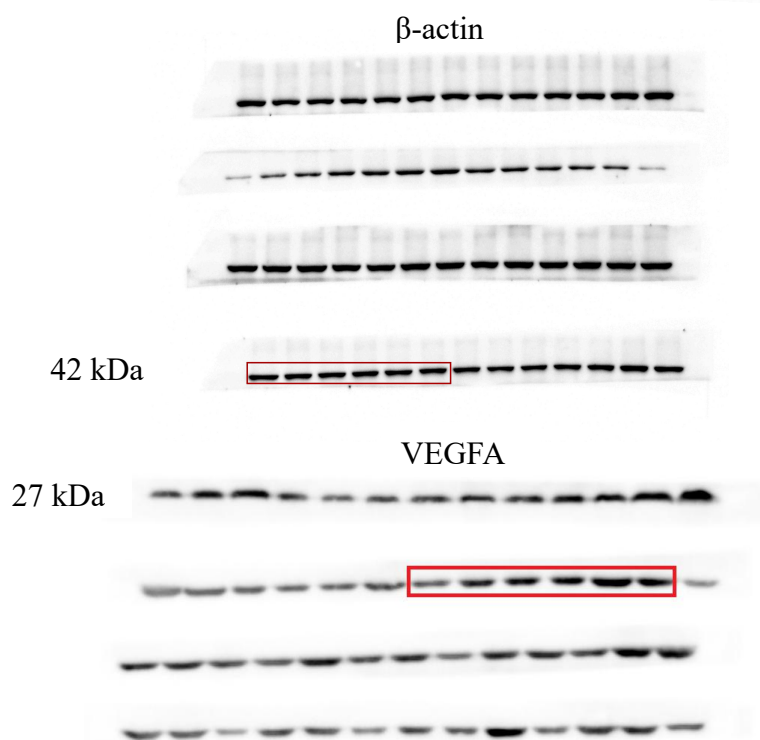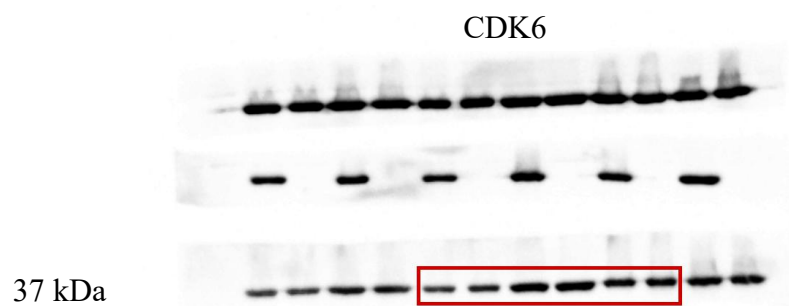

MYC

57kD

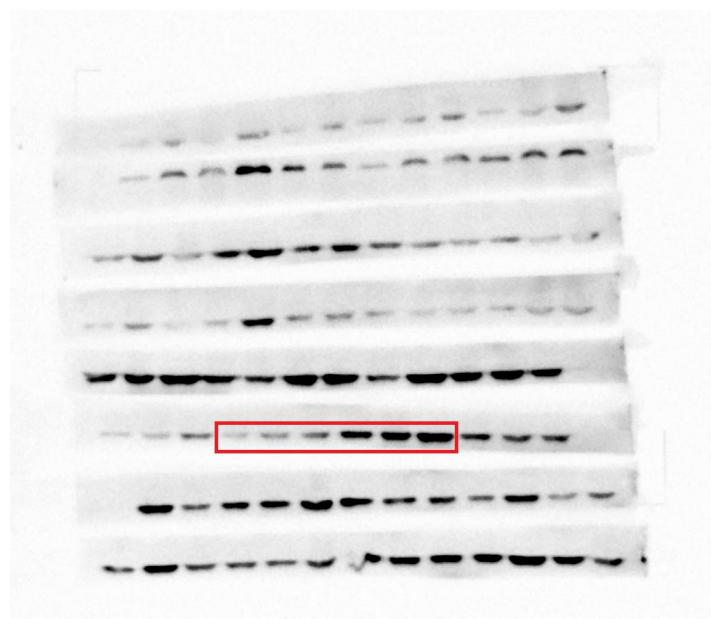

EGR1

58 kDa

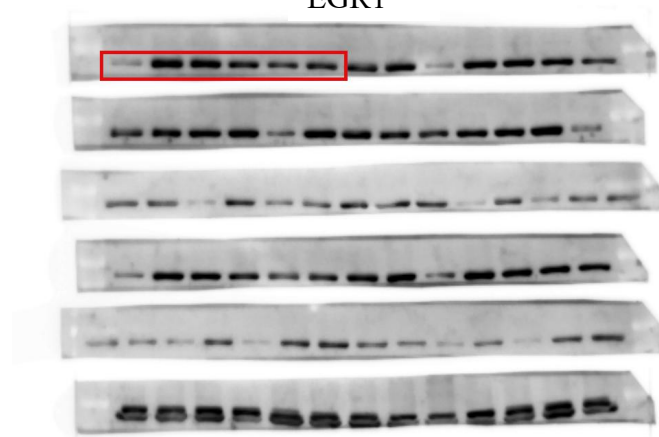

GAPDH

36 kDa

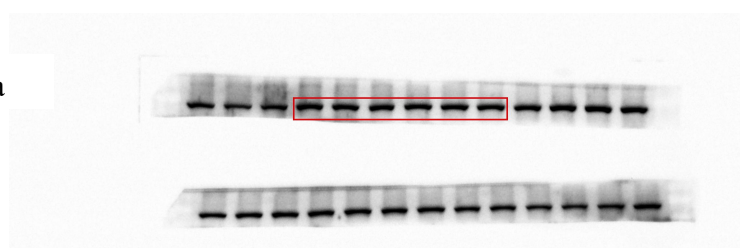

Figure 14

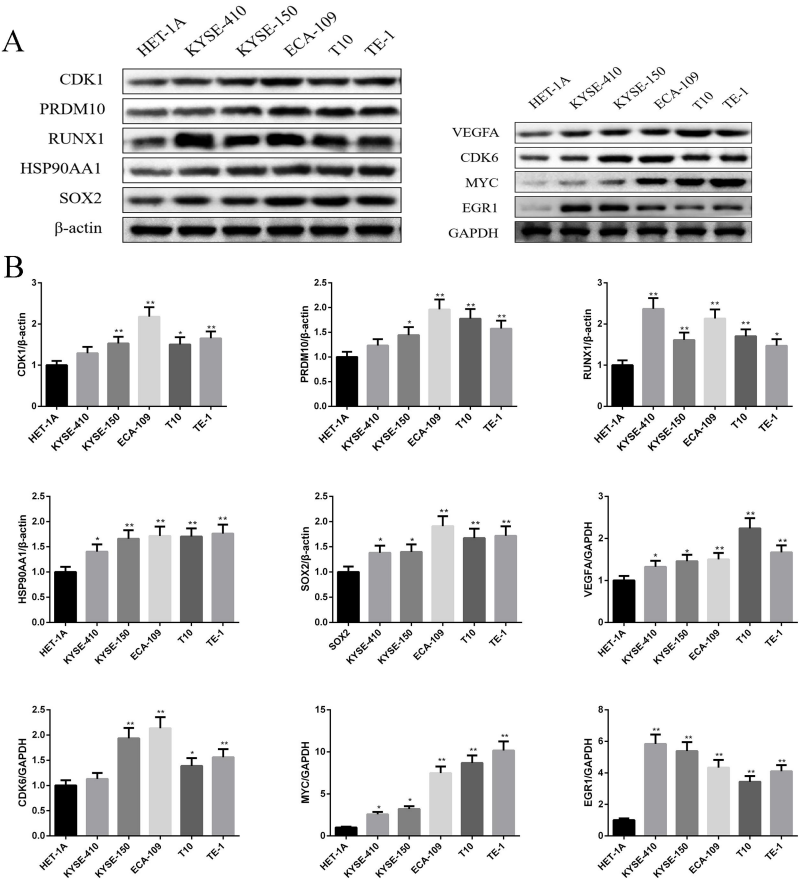

Supplement: Supplementary Figure S1 [file BSR-2020-1727_supp.pdf]
